# To Be Safe

**Published:** 1857-06

**Authors:** 


### TO BE SAFE,

BE in proper places at proper times, and mind your own business. With such restrictions, we believe human life is as safe in New York as in any other city on the globe. Nine times out of ten, the reports of persons who have fared badly, or who have fallen into the hands of the Philistines, indicate one of two things—an out-of-the-way place, or an unseasonable hour. A young man attends a private party, leaves at two o'clock in the morning, and is heard of no more. Another visits New York, and, with his pockets full of money, promenades the river streets alone, an hour or two after dark. It is at one o'clock in the morning that a man was "*knocked down, and robbed of several thousand dollars*," with no trace of the robbers—unless you get him to tell where and what he was doing between the hours of decent bed-time and those of early morning. The fact is, a good many of the robberies fathered on large cities never took place. The assignation and the gaming table are the maelstroms of half the "lost pocket-books" of the city morning newspapers. In case of a "respectable" citizen being knocked down and settled at two o'clock in the morning, the police daily report the transaction as having occurred "last evening," which, on more minute investigation, will be found to have been not long "*before day.*"

From long observation of city life, we instinctively set a man down as a loafer, or rowdy, or a loose character, the moment we see his name associated with a "loss" or a "knock-down;" and we maintain the position until we have conclusive proof to the contrary. We never hear of such men losing their pocket-books, or of being clubbed or garroted, as William B. Astor, Peter Cooper, Hamilton Fish, Washington Irving, Erastus Brooks, Governor Bradish, and others of equal standing. The fact is, respectable men—men of influence and position—are domestic men; they spend their evenings at home amid their families, or in attention to the necessary duties of good citizenship. It is the man himself, and not "*the city,*" that is the real father of the assaults and losses reported to have occurred from time to time. Not seldom are they men who have been intrusted with funds to pay out for other people; not always so, certainly, but this we do know, that if a man is courteous, minds his own business, and keeps good hours, he may walk the streets of every city in Christendom, and never meet a loss or receive a blow.

